# **Relationship between Hyperactivity of Depressor Septi Nasi Muscle and Changes of Alar Base and Flaring during Smile**

**Published:** 2016-01

**Authors:** Arash Beiraghi-Toosi, Ezatollah Rezaei, Elham Zanjani

**Affiliations:** 1 Department of Plastic Surgery, Endoscopic and Minimally Invasive Surgery Research Center, Mashhad University of Medical Sciences, Mashhad, Iran;; 2 Department of Plastic Surgery, Surgical Oncology Research Center, Mashhad University of Medical Sciences, Mashhad, Iran;; 3 Student Research Committee, Faculty of Medicine, Mashhad University of Medical Sciences, Mashhad, Iran

**Keywords:** Depressor septi nasi muscle, Hyperactivity, Alar base, Flaring, Smile

## Abstract

**BACKGROUND:**

Hyperactivity of depressor septi nasi muscle leads to smiling deformity and nasal tip depression. Lateral fascicles of this muscle help in widening the nostrils. The purpose of this study was to evaluate the relationship between the nasal length changes and the alar base and the alar flaring changes during smile.

**METHODS:**

Standard photographs are performed in the face and lateral views with forward gaze in the repose and maximum smile. Nasal length, alar base, and alar flaring were measured on the prints of the photographs. To decrease possible errors in the size of the printed photographs, middle face height from glabella to ANS was measured in the lateral view and the interpupil distance in the face view to standardize the measurements.

**RESULTS:**

Fifty cases were enrolled in this study. In 39 cases (78%), the nasal length was increased during smile. Forty-six cases (92%) had an increase in alar base diameter during smile. Alar flaring during smile increased in 48 cases (96%). Nasal length and alar base changes during smiling were not significantly correlated. Nasal length and alar flaring changes during smiling were not significantly related too. On the other hand, alar base and alar flaring changes during smile showed correlation. Alar base and alar flaring changes during smile were not significantly different in hyperactive and non-hyperactive cases.

**CONCLUSION:**

Nasal length change during smiling and hypertrophy of the medial fascicles of depressor septi nasi were not related to alar base or alar flaring change during smile.

## INTRODUCTION

Study on the anatomy of the nasolabial area muscles is becoming important. This is due to their importance in rhinoplasty for coordination of the nose and upper lip; especially during smile.^1^ During rhinoplasty, dynamic changes of the nose should be noted. Rhinoplasty is called an expression operation of the nose.^2^

Depressor septi nasi muscle is an important and effective muscle in nasal dynamics. It is often considered as a part of the alar segment of the nasalis muscle (dilatator naris). It is located deep to the upper lip mucosa. It depresses nasal septum and widens the nostrils together with the alar part of the nasalis muscle.^3^ It is composed of 3 fascicle groups including medial fascicles originating from anterior nasal spine and inserting to upper lip, intermediate fascicles locating between medial and lateral fascicles, and lateral fascicles that originate from maxilla and insert to alar cartilage.^4^ In Negroid noses, this muscle is more prominent and widens the wings^1^ and shortens the columella.^5^ To our knowledge, there is no study about the relationship of activities of different fascicles of this muscle. 

Hyperactivity of the medial fascicles of this muscle leads to the smiling deformity. This deformity consists of depression of nasal tip, shortening of upper lip, and a horizontal crease in upper lip.^6^ Great attention is paid to the resection or weakening of this muscle.^1^^,^^6^^-^^10^ In negroid noses, lateral fascicles can be released and redirected centrally to narrow the nostril base.^1^ In this study, we evaluated whether hyperactivity of the medial fasicles of this muscle and depression of nasal tip is associated with changes of alar base or alar flaring during smile. Any relationship between nasal length and alar base changes, if at all present, can have some clinical implications. 

## MATERIALS AND METHODS

The study was performed on 50 cases more than 18 years old with no history of rhinoplasty, cleft lip, incision or operation over upper lip. No selection was done regarding the activity of the depressor septi nasi muscle. The study was approved by the local ethics committee. Standard photographs were taken in the face and lateral views with forward gaze in the repose and maximum smile with the neck in the neutral position with digital camera with LED screen with maximum zoom from hairline to chin. 

**Fig. 1:**
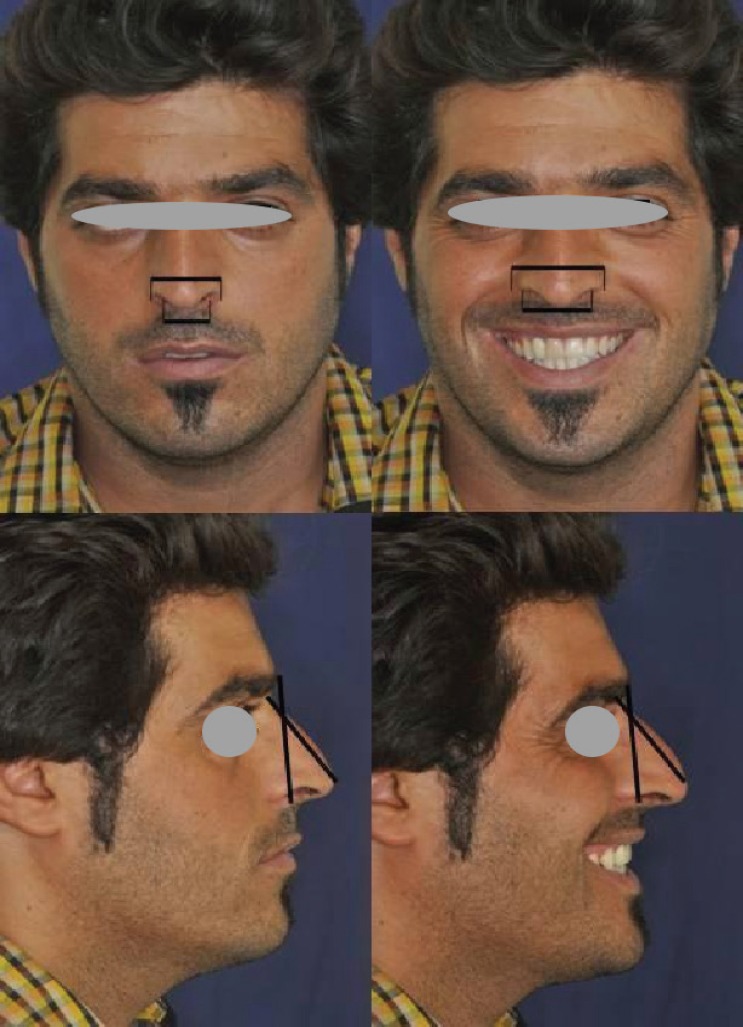
Measurements on face and lateral photographs in the repose and maximum smile

Nasal length was measured from radix to nasal tip on the prints of the lateral photographs. Alar base (the distance between the points of the junction of alar base to upper lip on both sides) and alar flaring (the maximum distance between the most lateral points of the alar base convexity on both sides) were measured on the prints of the face photographs. To increase reliability, 2 measurements were done and their average was calculated. To decrease possible errors due to minor differences in the scales of the printed photographs and positional artifacts, middle face height from glabella to subnasale in the lateral view and the interpupil distance in the face view were measured to standardize the measurements (Figure 1).

## RESULTS

Fifty cases were enrolled in this study. Forty five cases (90%) were female and five (10%) were male. The mean age was 25.5±7.02 years (min=18, max=50 years). The mean change of the nasal length with smiling was 2.2±2.70 mm (min=-3.0, max=9.4 mm). This was 4.7±5.94 percent of the nonsmiling nasal length. In 39 cases (78%), the nasal length increased during smiling. The mean increase in nasal length with smile in this group was 2.1±2.20 mm. In 10 cases (20%), nasal length decreased with smile. The mean decrease in this group was 1.4±0.98 mm. In 1 case (2%), there was no change of nasal length during smiling.

The mean change of alar base during smiling was 4.0±2.69 mm (min=-2.1, max=9.0 mm). It was 20.91±15.561 percent of the nonsmiling nasal base. Forty-six cases (92%) had the increase of the alar base diameter during smiling. In this group, the mean increase of alar base was 4.4±2.40 mm. In 2 cases (4%), alar base diameter decreased during smiling (2.1 mm and 0.3 mm). In 2 other cases (4%), alar base did not change during smiling.

The mean alar flaring change with smile was 3.8±1.62 mm (min=-0.1, max=7.4 mm). This change was 10.87±4.787 percent of the nonsmiling alar flaring. Alar flaring during smiling increased in 48 cases (96%). In this group, the mean alar flaring increase was 1.0±1.44 mm. Only 1 case had a decreased alar flaring (0.1 mm) and 1 case showed no change.

**Fig. 2a-2f:**
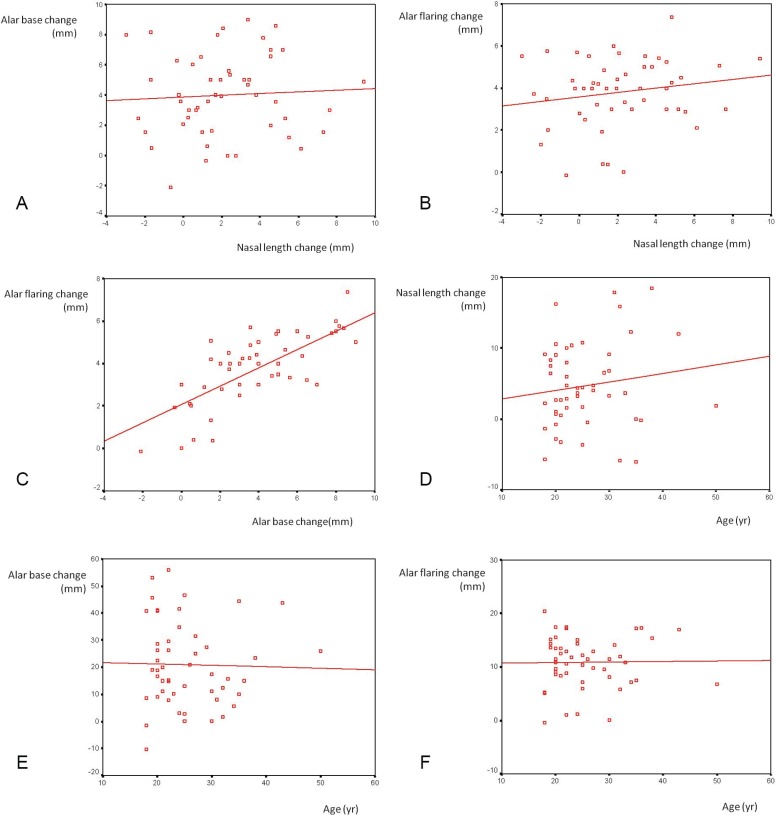
The diagram of relationship between nasal length and alar base changes during smiling

Nasal length and alar base changes during smiling were not significantly related statistically (Pearson=0.056, *p*=0.697). The percent of the nasal length and alar base changes during smiling were not significantly related, too (Pearson=0.109, *p*=0.453) (Figure 2a). Nasal length and alar flaring changes during smiling were not significantly related statistically (Pearson=0.175, *p*=0.223). The percent of the nasal length and alar flaring changes with smile were not related significantly (Pearson=0.127, *p*=0.378) (Figure 2b).

On the other hand, alar base and alar flaring changes during smiling were related significantly (Pearson=0.716, *p*=0.001). The percent of alar base and alar flaring changes during smiling were significantly related (Pearson=0.657, *p*<0.001) (Figure 2c). The mean change of alar base during smiling was 3.7±3.28 mm in those with decreased nasal length and 4.1±2.58 mm in those with increased nasal length during smiling. This was not statistically significant (t=-0.378, df=47, *p*=0.707). The mean change of alar flaring in those with decreased nasal length during smiling was 3.6±1.97 and in those with increased nasal length was 3.9±1.56 mm. This was not statistically significant (t=-0.536, df=47, *p*=0.595). 

The age of the cases was not statistically related to the percent of alar length change during smiling (Pearson=0.142, *p*=0.324). The age of the cases was not statistically related to the percent of alar base change during smiling, too (Pearson=-0.024, *p*=0.871). The age was not statistically related to the percent of alar flaring change during smiling (Pearson=0.015, *p*=0.918).

If we split our cases to a group with nasal lenghtening during smile (as the group with depressor septi nasi hyperactivity) and another group without nasal lenghtening during smile (as the group without depressor septi nasi hyperactivity), the mean increase in alar base during smile in the hyperactive group was 4.1±2.58 mm and in the non-hyperactive group was 3.6±3.15 mm. This difference was not significant (Mann-Whitney U=195.5, *p*=0.656). The mean increase in alar flaring during smile in the hyperactive group was 3.9±1.56 mm and in the non-hyperactive group was 3.5±1.89 mm. This difference was not significant (Mann-Whitney U=193.0, *p*=0.614) (Figures 2d-2f).

## DISCUSSION

In a study done by Rohrich, depressor septi nasi was attached to orbicularis oris in 62% of cadavers and to the periosteom in 22% and totally in 84% of cases the muscle was visible and detectable.^6^ In our study, the nasal length was increased in 78% of cases during smiling. The methods of these two studies are not the same. The first study is an anatomic study on cadavers whereas our study is a clinical study on healthy people. In general, it seems that in many cases that the muscle is visible and detectable, the nasal length increases during smiling.

This high prevalence of the nasal length increase during smile, obtained in our study, should be clinically examined widely. Ethnic factors affect its prevalence. For example, in the study done by Tellioglu in Turkey, 36% of cases had hyperdynamic nasal tip related to depressor septi nasi hyperactivity.^8^ In Japanese ethnicity, this muscle is very rudimentary.^11^ In our clinical study, 78% cases had increase of nasal length with smile. Study in varied ethnic populations will clarify its prevalence in different ethnicities.

Study of nasal length and alar base diameter during repose and smile before and after rhinoplasty is previously presented by.^12^ The changes of these diameters during operation have important effects on the results. Alar base change during smile is affected by the function of different muscles. Levator labii superioris alaeque nasi, alar and transverse parts of the nasalis muscle, and musculus dilator naris are among the most important muscles affecting alar base. Levator labii superioris alaeque nasi elevates alar base and depressor septi nasi has a role in widening it.^1^^,^^13^^-^^16^

Ideally, to evaluate one muscle, we should omit other muscles. In an individual case, we cannot clinically determine the affect of every individual muscle on the nasal base. To be able to perform this study practically, it is assumed that various activities of the affecting muscles other than lateral fascicles of depressor septi nasi are distributed among the studied cases and, statistically, the study of the relationship of nasal length, alar base, and alar flaring is not affected by other confounding variables.

In our study, nasal length change during smiling was not related to alar base or alar flaring during smiling. It may imply that hypertrophy of the medial fascicles of depressor septi nasi muscle is not related to alar base or alar flaring change during smiling. Based on this result, we may conclude that manipulation of medial fascicles of this muscle during rhinoplasty does not affect the alar base and alar flaring. More interventional studies with larger sample size are needed to validate this assumption.

Lateral fascicles of this muscle have a role in alar base width. Studies on the interaction of different muscles acting on the alae can clarify the effects of varied interventions on the muscles during dynamic rhinoplasty. In our study, in 92% of cases, alar base increased during smiling. This increase is a common complaint of pateints that undergo the rhinoplasty operation. Our study shows that the exicision of depressor septi nasi may not change this increase in alar base diameter. In our study, in 96% of cases, alar flaring increased during smiling, too, and there was a significant relationship between the change of the alar base and the alar flaring during smiling. Alar base excision in patients with severe alar flaring (more than 2 mm) is a proper technique during rhinoplasty. But this is rather a static change in the nose than a dynamic change for decreasing smile effects.

It was shown that depressor septi nasi hypertrophy can be a mechanism for shortening of columella in the Negroid ethnicity.^5^ In Negroid noses, alar base is wide and alar flaring is severe. This finding has been attributed to the hypertrophy of the lateral fascicles of the depressor septi nasi.^1^ Further studies about the interaction of the nasal muscles may yield interesting results in this ethnicity. Nasal length change during smiling and hypertrophy of the medial fascicles of depressor septi nasi muscle is not related to alar base or alar flaring change during smiling. 
